# Supplementary material for: Analysis of Leukocyte Subpopulations by Flow Cytometry during Hospitalization Depending on the Severity of COVID-19 Course
Source: Biomedicines. 2023 Oct 8;11(10):2728. doi: 10.3390/biomedicines11102728 (PMC10604221; doi:10.3390/biomedicines11102728)
Supplement: Supplementary file 1 [file biomedicines-11-02728-s001.zip › biomedicines-2599179-supplementary.pdf]

biomedicines

Supplementary Material

The cells subpopulation results of flow cytometry depending on the severity of the COVID-19 course

Elżbieta Rutkowska[1*], Iwona Kwiecień[1], Ewa Pietruszka[2], Piotr Rzepecki[3], Karina Jahnz-Różyk[2]

*** Correspondence:** Elżbieta Rutkowska erutkowska@wim.mil.pl

1 Supplementary Table

biomedicines

MDPI

Table S1. Changes in flow cytometry results during hospitalization and during the visit 3 months after hospitalization

Parameter	The course of COVID	n	Results in T0					Results in T1					T1 - T0
			Mean	SD	Median	Q1	Q3	Mean	SD	Median	Q1	Q3	* p< 0.05
WBC	non-severe	10	7821.0	2431.8	8060.0	5507.5	9672.5	6002.0	1525.0	5785.0	5007.5	6502.5	0.067
	severe	7	5811.4	2955.5	4840.0	4030.0	5815.0	5965.7	1235.4	6060.0	5505.0	6540.0	0.901
lymphocytes	non-severe	10	25.2	14.2	21.1	16.0	28.3	34.3	9.5	34.6	26.9	38.4	0.085
	severe	7	34.4	9.5	37.3	33.3	39.1	30.5	5.0	29.3	27.6	31.0	0.282
T lymphocytes	non-severe	10	18.4	12.7	14.9	10.5	19.6	27.4	8.7	27.6	20.5	33.9	0.065
	severe	7	27.8	8.1	29.2	26.7	31.0	24.6	5.1	23.5	21.5	25.8	0.271
CD4 T cells	non-severe	10	12.0	9.5	7.6	6.1	13.6	15.8	4.8	15.1	13.6	17.8	0.189
	severe	7	17.9	6.1	19.1	16.2	20.9	13.3	3.3	13.4	11.9	15.5	0.063
CD8 T cells	non-severe	10	5.8	3.4	6.1	3.3	6.6	10.9	5.6	9.1	6.4	13.4	*0.019
	severe	7	9.0	6.3	7.5	4.9	10.9	10.9	5.7	10.1	8.4	12.6	0.094
CD4/CD8	non-severe	10	2.2	1.0	2.3	1.4	3.0	1.7	0.8	1.5	1.0	2.6	*0.002
	severe	7	2.9	2.2	2.6	1.2	4.1	1.8	1.7	1.5	0.8	1.8	0.067
natural killer cells	non-severe	10	3.2	1.6	2.9	2.2	4.4	4.0	1.4	3.8	2.7	5.2	0.070
	severe	7	3.0	1.4	3.0	2.0	4.1	3.6	1.5	3.1	2.7	3.8	0.406

Parameter	The course of COVID	n	Results in T0					Results in T1					T1 - T0
			Mean	SD	Median	Q1	Q3	Mean	SD	Median	Q1	Q3	* p< 0.05
B lymphocytes	non-severe	10	3.7	2.0	2.7	2.3	4.6	2.8	1.3	2.8	2.2	3.1	0.062
	severe	7	3.6	1.5	3.2	2.7	4.4	2.2	0.8	2.1	1.9	2.8	0.098
neutrophils	non-severe	10	66.9	16.2	71.0	64.6	77.5	55.6	8.9	55.2	50.0	64.3	*0.041
	severe	7	55.5	11.0	55.5	50.2	57.5	59.3	6.0	58.8	57.7	63.6	0.388
eosinophils	non-severe	10	1.0	1.7	0.2	0.1	0.9	3.1	2.6	2.0	1.7	3.8	*0.002
	severe	7	3.5	2.5	3.7	2.0	4.3	3.1	1.4	3.4	2.5	3.8	0.604
basophils	non-severe	10	0.4	0.3	0.3	0.2	0.6	0.7	0.3	0.8	0.4	0.9	*0.028
	severe	7	0.9	0.6	1.2	0.5	1.4	0.6	0.5	0.6	0.2	0.8	0.187
monocytes	non-severe	10	6.8	1.7	6.5	5.9	6.7	6.3	1.5	6.1	5.7	6.9	0.527
	severe	7	6.0	2.0	6.0	4.7	7.8	6.7	1.5	6.6	6.0	7.8	0.332
CD16+ monocytes	non-severe	10	11.0	7.6	7.4	6.4	15.4	15.2	6.9	15.7	11.8	20.3	0.316
	severe	7	16.6	7.6	15.6	11.7	17.2	18.2	7.1	18.6	16.5	23.2	0.624
Lymphocytes T (%/L)	non-severe	10	70.1	8.9	69.7	67.2	74.1	79.2	7.0	80.4	75.1	83.2	*0.002
	severe	7	80.6	4.0	80.0	78.3	82.8	80.5	6.8	81.9	80.2	85.0	0.970
Lymphocytes T CD4+ (%/L)	non-severe	10	44.1	10.8	42.1	38.8	49.9	46.0	8.6	45.3	40.0	53.0	0.339
	severe	7	52.0	12.3	51.3	41.8	62.7	44.0	10.9	47.7	35.7	53.4	*0.017
Lymphocytes T CD8+ (%/L)	non-severe	10	23.3	8.3	23.0	15.9	28.7	30.7	10.6	29.6	21.8	36.3	<0.001

Parameter	The course of COVID	n	Results in T0					Results in T1					T1 - T0
			Mean	SD	Median	Q1	Q3	Mean	SD	Median	Q1	Q3	* p< 0.05
	severe	7	26.1	13.6	23.5	16.0	35.1	35.0	14.5	32.5	30.3	44.9	*0.011
NK cells (%/L)	non-severe	10	14.4	9.2	12.2	9.8	12.7	12.4	5.9	10.5	7.8	15.0	0.269
	severe	7	8.8	3.1	7.8	6.9	11.2	12.1	5.5	9.9	8.6	13.5	0.116
Lymphocytes B (%/L)	non-severe	10	15.4	5.2	14.6	12.3	19.5	8.4	2.6	9.0	7.8	9.7	*0.002
	severe	7	10.5	3.3	11.0	8.0	12.4	7.6	2.5	7.3	6.3	9.5	0.108
T lymphocytes (k/ul)	non-severe	10	1249.0	454.6	1269.6	1082.8	1377.3	1668.3	865.4	1446.4	1161.3	1874.8	0.211
	severe	7	1589.5	987.2	1248.4	1208.1	1448.5	1462.4	419.3	1339.3	1199.5	1616.7	0.774
CD4+ lymphocytes (k/ul)	non-severe	10	789.7	364.0	674.0	545.4	996.3	949.6	421.1	780.9	730.2	1040.1	0.370
	severe	7	1097.3	930.4	767.8	650.4	1021.1	770.0	157.9	830.8	738.9	868.2	0.383
CD8+ lymphocytes (k/ul)	non-severe	10	415.3	188.2	398.7	308.5	570.1	672.4	486.4	452.4	379.2	849.3	0.106
	severe	7	449.6	220.3	377.5	321.1	562.5	663.7	396.7	569.5	419.8	858.8	0.064
NK cells (k/ul)	non-severe	10	233.9	143.9	217.0	179.3	233.9	244.2	107.6	241.1	151.2	328.5	0.813
	severe	7	153.9	49.7	169.7	115.5	195.0	215.8	94.9	197.8	152.9	259.4	0.205
B lymphocytes (k/ul)	non-severe	10	262.9	99.9	238.8	212.3	310.8	175.3	101.8	134.3	123.6	214.4	*0.007
	severe	7	228.6	224.4	128.6	117.3	197.8	134.8	54.3	163.8	88.3	175.6	0.350

Table S2. Changes in flow cytometry results during the visit 3 months after hospitalization (T1) and during the visit 6 months after hospitalization (T2).

Parameter	The course of COVID	n	Results in T1					Results in T2					T2 - T1
			Mean	SD	Median	Q1	Q3	Mean	SD	Median	Q1	Q3	* p< 0.05
WBC	non-severe	14	6055.0	1750.2	5785.0	4825.0	6592.5	6885.0	2690.6	5860.0	5645.0	7337.5	0.108
	severe	3	7640.0	6833.2	4490.0	3720.0	9985.0	6606.7	553.0	6540.0	6315.0	6865.0	0.805
lymphocytes	non-severe	14	34.8	11.7	32.9	26.9	42.8	37.3	12.7	42.3	28.4	46.6	0.401
	severe	3	27.4	16.2	25.1	18.8	34.9	27.4	2.1	27.8	26.5	28.6	1,000
T lymphocytes	non-severe	14	27.7	10.1	25.3	20.5	34.2	29.3	11.2	31.5	22.3	37.1	0.582
	severe	3	20.2	14.8	21.7	13.2	28.0	20.7	3.1	19.1	18.9	21.7	0.958
CD4 T cells	non-severe	10	12.0	9.5	7.6	6.1	13.6	15.8	4.8	15.1	13.6	17.8	0.189
	severe	7	17.9	6.1	19.1	16.2	20.9	13.3	3.3	13.4	11.9	15.5	0.063
CD8 T cells	non-severe	14	10.0	5.2	9.1	6.0	13.2	10.3	5.3	8.8	6.1	14.3	0.874
	severe	3	7.1	4.5	8.6	5.3	9.6	8.3	1.7	8.7	7.6	9.2	0.763
CD4/CD8	non-severe	14	1.9	0.7	1.9	1.1	2.7	2.0	0.8	2.0	1.4	2.2	0.834
	severe	3	1.6	0.3	1.5	1.5	1.8	1.4	0.5	1.7	1.3	1.7	0.438
natural killer cells	non-severe	14	3.9	1.4	3.7	2.6	4.7	4.1	1.9	3.7	3.0	4.6	0.568
	severe	3	5.5	3.3	4.7	3.7	6.9	5.7	3.3	5.6	4.0	7.3	0.938

Parameter	The course of COVID	n	Results in T1					Results in T2					T2 - T1
			Mean	SD	Median	Q1	Q3	Mean	SD	Median	Q1	Q3	* p< 0.05
B lymphocytes	non-severe	14	3.2	1.6	2.8	2.3	4.3	3.9	1.7	4.0	3.0	5.0	0.148
	severe	3	1.8	1.2	1.4	1.1	2.3	1.1	0.6	1.1	0.8	1.4	0.317
neutrophils	non-severe	14	56.3	11.5	59.4	48.6	64.1	52.5	14.3	47.8	45.3	60.6	0.227
	severe	3	66.4	18.6	70.3	58.2	76.5	61.8	4.1	59.7	59.5	63.2	0.762
eosinophils	non-severe	14	2.5	1.8	2.0	1.6	2.6	2.2	1.5	2.0	1.1	3.3	0.489
	severe	3	1.8	1.3	1.7	1.2	2.5	1.4	1.1	1.5	0.9	2.0	0.636
basophils	non-severe	14	0.7	0.3	0.7	0.5	0.9	0.7	0.5	0.7	0.5	0.9	0.791
	severe	3	0.2	0.2	0.1	0.1	0.3	0.6	0.5	0.6	0.4	0.9	0.195
monocytes	non-severe	14	5.8	1.4	6.0	5.3	6.8	6.8	2.2	7.2	4.9	9.0	0.054
	severe	3	5.1	0.9	5.2	4.7	5.6	8.7	2.0	8.9	7.8	9.8	0.160
CD16+ monocytes	non-severe	14	14.9	8.8	15.5	10.0	20.3	9.2	5.4	10.4	4.9	11.2	0.084
	severe	3	13.3	9.2	12.3	8.5	17.6	10.3	4.8	9.4	7.7	12.5	0.712
Lymphocytes T (%/L)	non-severe	14	78.9	5.3	79.0	75.1	81.4	77.0	6.4	76.8	71.5	81.6	0.397
	severe	3	66.8	25.8	76.5	57.1	81.5	75.6	11.8	75.6	69.7	81.5	0.421
Lymphocytes T CD4+ (%/L)	non-severe	14	48.4	6.3	49.1	41.7	53.1	48.1	7.4	47.9	43.3	51.6	0.831

Parameter	The course of COVID	n	Results in T1					Results in T2					T2 - T1
			Mean	SD	Median	Q1	Q3	Mean	SD	Median	Q1	Q3	* $p < 0.05$
	severe	3	39.9	15.7	47.8	34.8	48.9	42.7	11.6	43.5	37.1	48.7	0.538
Lymphocytes T CD8+ (%/L)	non-severe	14	27.7	8.4	24.8	21.4	36.0	26.9	8.0	24.5	20.1	34.7	0.731
	severe	3	24.5	9.2	23.7	19.8	28.9	29.9	4.1	31.3	28.3	32.2	0.472
NK cells (%/L)	non-severe	14	11.6	3.9	10.8	9.8	14.2	12.3	6.4	10.8	8.0	15.3	0.641
	severe	3	22.7	13.6	20.3	15.4	28.8	20.6	11.2	22.4	15.5	26.6	0.486
Lymphocytes B (%/L)	non-severe	14	9.4	3.3	9.0	7.7	11.1	10.7	4.6	9.6	8.5	11.8	0.365
	severe	3	10.5	12.7	3.2	3.2	14.2	3.8	1.8	4.0	3.0	4.8	0.416
T lymphocytes (k/ul)	non-severe	14	1696.9	856.2	1547.9	1161.3	2048.4	1891.0	778.1	1844.1	1331.0	2066.0	0.447
	severe	3	967.8	493.7	727.6	683.9	1131.6	1365.6	213.8	1344.5	1253.9	1466.9	0.422
CD4+ lymphocytes (k/ul)	non-severe	14	1006.5	410.9	953.2	738.0	1266.6	1156.0	428.3	1111.9	949.2	1426.8	0.318
	severe	3	583.5	327.6	418.0	394.8	689.4	766.0	186.5	669.9	658.5	825.5	0.557
CD8+ lymphocytes (k/ul)	non-severe	14	637.5	466.5	474.7	363.5	738.4	678.2	377.6	591.7	464.8	834.8	0.714
	severe	3	346.4	115.6	309.6	281.7	392.8	552.1	154.5	569.0	479.4	633.2	0.298
NK cells (k/ul)	non-severe	14	232.9	104.0	213.6	139.9	300.9	273.0	126.4	237.4	162.2	361.7	0.119
	severe	3	404.3	325.5	408.6	242.6	568.1	381.7	247.6	341.0	249.0	494.1	0.704

Parameter	The course of COVID	n	Results in T1					Results in T2					T2 - T1
			Mean	SD	Median	Q1	Q3	Mean	SD	Median	Q1	Q3	* p< 0.05
B lymphocytes (k/ul)	non-severe	14	188.5	93.2	162.0	123.6	267.5	247.7	104.1	235.5	178.0	332.1	0.039
	severe	3	188.8	252.9	62.9	43.2	271.4	72.5	42.3	71.9	51.2	93.5	0.455

Table S3. Changes in flow cytometry results during hospitalization (T0) and during the visit 6 months after hospitalization (T2)

Parameter	The course of COVID	n	Results in T0					Results in T2					T2 – T0
			Mean	SD	Median	Q1	Q3	Mean	SD	Median	Q1	Q3	*p< 0.05
WBC	non-severe	11	8730.9	3338.9	8900.0	6425.0	10190.0	6648.2	1318.3	6110.0	5735.0	6995.0	*0.036
	severe	2	7665.0	2566.8	7665.0	6757.5	8572.5	6045.0	1789.0	6045.0	5412.5	6677.5	0.208
lymphocytes	non-severe	11	24.6	13.8	22.4	14.5	26.5	37.7	11.1	43.9	29.8	46.3	*0.006
	severe	2	48.7	16.2	48.7	42.9	54.4	41.4	2.6	41.4	40.4	42.3	0.586
T lymphocytes	non-severe	11	18.1	12.3	16.6	9.2	19.1	29.5	8.7	32.5	23.8	35.0	*0.006
	severe	2	39.6	14.4	39.6	34.5	44.6	34.2	3.9	34.2	32.8	35.5	0.599
CD4 T cells	non-severe	11	12.6	9.0	10.3	6.3	14.5	19.1	5.2	20.0	16.9	22.1	*0.014
	severe	2	19.5	3.6	19.5	18.2	20.7	18.7	1.1	18.7	18.3	19.1	0.860

Parameter	The course of COVID	n	Results in T0					Results in T2					T2 – T0
			Mean	SD	Median	Q1	Q3	Mean	SD	Median	Q1	Q3	*p< 0.05
CD8 T cells	non-severe	11	4.8	3.4	3.8	2.7	5.7	9.5	4.3	8.2	5.8	13.3	*0.002
	severe	2	18.2	9.0	18.2	15.0	21.3	14.9	5.0	14.9	13.1	16.6	0.448
CD4/CD8	non-severe	11	2.7	0.9	2.6	2.4	3.2	2.3	0.8	2.0	1.8	3.0	*0.009
	severe	2	1.2	0.4	1.2	1.0	1.3	1.4	0.5	1.4	1.2	1.5	0.295
natural killer cells	non-severe	11	2.9	2.1	2.7	1.7	4.0	4.6	2.1	4.1	3.0	5.9	*0.030
	severe	2	4.2	0.1	4.2	4.2	4.3	3.6	0.8	3.6	3.3	3.9	0.442
B lymphocytes	non-severe	11	3.6	1.7	3.3	2.5	4.3	3.5	1.3	3.7	3.0	4.4	0.962
	severe	2	4.9	2.0	4.9	4.2	5.6	3.6	0.4	3.6	3.5	3.8	0.584
neutrophils	non-severe	11	67.8	16.2	70.2	64.9	78.4	52.8	12.5	49.1	45.4	61.7	*0.003
	severe	2	40.7	14.4	40.7	35.6	45.7	48.7	0.1	48.7	48.6	48.7	0.574
eosinophils	non-severe	11	0.4	0.6	0.2	0.2	0.4	2.1	1.2	1.7	1.3	2.5	<0.001
	severe	2	1.9	0.2	1.9	1.8	1.9	1.6	0.6	1.6	1.4	1.8	0.500
basophils	non-severe	11	0.4	0.2	0.4	0.3	0.5	0.6	0.4	0.6	0.3	0.9	0.077
	severe	2	1.5	0.5	1.5	1.3	1.6	0.9	0.4	0.9	0.8	1.1	0.553
monocytes	non-severe	11	7.2	2.6	6.5	5.6	7.4	6.3	2.2	5.9	4.5	8.1	0.391

Parameter	The course of COVID	n	Results in T0					Results in T2					T2 – T0
			Mean	SD	Median	Q1	Q3	Mean	SD	Median	Q1	Q3	*p< 0.05
	severe	2	7.0	3.2	7.0	5.8	8.1	7.5	2.7	7.5	6.6	8.5	0.361
CD16+ monocytes	non-severe	11	10.0	7.1	6.9	5.6	10.7	8.7	5.3	10.3	3.7	11.1	0.691
	severe	2	20.7	2.8	20.7	19.7	21.6	17.7	2.1	17.7	16.9	18.4	0.105
Lymphocytes T (%/L)	non-severe	11	70.9	9.4	70.2	66.5	74.9	78.4	4.2	77.9	76.8	82.0	*0.016
	severe	2	80.8	2.6	80.8	79.8	81.7	82.5	4.3	82.5	80.9	84.0	0.391
Lymphocytes T CD4+ (%/L)	non-severe	11	48.2	10.3	45.8	39.9	55.6	51.8	6.6	51.2	48.1	56.4	0.169
	severe	2	41.0	6.2	41.0	38.8	43.2	45.5	5.6	45.5	43.5	47.4	0.064
Lymphocytes T CD8+ (%/L)	non-severe	11	19.0	5.4	16.3	15.2	21.8	24.6	6.4	25.8	18.9	27.1	<0.001
	severe	2	36.1	6.4	36.1	33.8	38.3	35.7	10.0	35.7	32.1	39.2	0.899
NK cells (%/L)	non-severe	11	13.1	9.2	12.3	7.9	12.8	12.1	4.0	12.7	8.9	14.6	0.620
	severe	2	9.3	3.3	9.3	8.1	10.4	8.8	2.7	8.8	7.9	9.8	0.500
Lymphocytes B (%/L)	non-severe	11	16.0	6.2	16.3	12.5	19.2	9.5	1.9	9.7	8.1	10.0	*0.006
	severe	2	10.0	0.7	10.0	9.8	10.3	8.8	1.6	8.8	8.2	9.3	0.587
T lymphocytes (k/ul)	non-severe	11	1412.5	668.8	1384.5	962.8	1784.0	1938.9	671.0	1800.5	1485.9	2230.9	0.087
	severe	2	3215.7	2115.4	3215.7	2467.8	3963.6	2099.2	846.0	2099.2	1800.0	2398.3	0.431

Parameter	The course of COVID	n	Results in T0					Results in T2					T2 – T0
			Mean	SD	Median	Q1	Q3	Mean	SD	Median	Q1	Q3	*p< 0.05
CD4+ lymphocytes (k/ul)	non-severe	11	963.4	472.9	1033.3	579.8	1317.6	1240.3	319.9	1196.6	1006.5	1486.0	0.123
	severe	2	1537.1	775.7	1537.1	1262.9	1811.4	1120.3	266.1	1120.3	1026.2	1214.4	0.454
CD8+ lymphocytes (k/ul)	non-severe	11	371.3	182.4	332.9	269.2	488.6	643.8	370.5	526.1	402.1	819.9	*0.020
	severe	2	1506.5	1154.2	1506.5	1098.4	1914.5	942.6	569.2	942.6	741.4	1143.8	0.403
NK cells (k/ul)	non-severe	11	266.1	321.6	190.6	138.8	233.7	297.4	126.0	328.2	177.0	395.4	0.738
	severe	2	320.1	97.0	320.1	285.8	354.4	210.0	13.1	210.0	205.4	214.7	0.315
B lymphocytes (k/ul)	non-severe	11	280.7	85.4	288.9	238.8	320.6	229.5	80.5	232.7	176.3	295.2	0.094
	severe	2	401.0	277.5	401.0	302.9	499.1	213.8	38.8	213.8	200.1	227.5	0.467
